# *Ex situ* and *in situ* characterization of patterned photoreactive thin organic surface layers using friction force microscopy

**DOI:** 10.1002/sca.21159

**Published:** 2014-09-02

**Authors:** Quan Shen, Matthias Edler, Thomas Griesser, Astrid-Caroline Knall, Gregor Trimmel, Wolfgang Kern, Christian Teichert

**Affiliations:** 1 Institute of Physics, Montanuniversität LeobenLeoben, Austria; 2 Chair of Chemistry of Polymeric Materials, Montanuniversität LeobenLeoben, Austria; 3 Institute for Chemistry and Technology of Materials, NAWI Graz, Graz University of TechnologyGraz, Austria

**Keywords:** friction force microscopy, adhesion force, photolithography, photoreactive surface layers

## Abstract

Photolithographic methods allow an easy lateral top-down patterning and tuning of surface properties with photoreactive molecules and polymers. Employing friction force microscopy (FFM), we present here different FFM-based methods that enable the characterization of several photoreactive thin organic surface layers. First, three *ex situ* methods have been evaluated for the identification of irradiated and non-irradiated zones on the same organosilane sample by irradiation through different types of masks. These approaches are further extended to a time dependent *ex situ* FFM measurement, which allows to study the irradiation time dependent evolution of the resulting friction forces by sequential irradiation through differently sized masks in crossed geometry. Finally, a newly designed *in situ* FFM measurement, which uses a commercial bar-shaped cantilever itself as a noncontact shadow mask, enables the determination of time dependent effects on the surface modification during the photoreaction. SCANNING 36:590–598, 2014.

## Introduction

Rapid and economical fabrication of thin film devices based on organic semiconductors is becoming a major driving force in the development of photochemistry in polymer science (Yu and Van de Lagemaat, 2011). Photolithographic techniques enable an easy lateral patterning with sub-micrometer resolution and bear the potential of tuning surface properties such as polarity, surface friction, chemical reactivity, etc. in a wide range (Tarlov *et al*., 1993; Thompson *et al*., 1994; Hoefler *et al*., 2007; Griesser *et al*., 2007, 2008). Defined photoreactions of photoacid generator groups are successfully utilized in polymeric layers and can be transferred to self-assembled monolayers (SAM) (Marchl *et al*., 2010; Pacher *et al*., 2007, 2008; Ramil *et al*., 2012; Yamaguchi *et al*., 2008). As we have demonstrated previously, two dimensional patterns in functionality in a SAM can be created by photoligthography and subsequenty post-exposure reactions. Since different surface terminations have different adhesive interactions with the probe of an atomic force microscope (AFM), friction force microscopy (FFM) (Mate *et al*., 1987; Marti *et al*., 1990; Meyer and Amer, 1990), also called lateral force microscopy, may reveal the hierarchy of adhesion in such patterned surface layers (Hlawacek *et al*., 2009).

In our previous *ex situ* FFM characterizations of photogenerated patterns of spin casted thin films (Lex *et al*., 2008; Hlawacek *et al*., 2009; Hoefler *et al*., 2010), detecting height contrast in the simultaneously recorded topographic image was a challenging prerequisite for identifying different terminations of photopatterned surfaces. However, placing a contact mask directly on a thin surface layer may influence the height results of illuminated and non-illuminated stripes. In addition, photoreactions often cause no or only minor topographical changes without subsequent derivatization of the photoproduct. Considering these two factors, there is a need to develop new *ex situ* procedures, which allows clear identification of illuminated areas without the assistance of height contrast in the topographical image. On the other hand, to detect the real height change after illumination, a non-contact mask which is positioned close to the surface should be used in an *in situ* illumination FFM measurement.

In this contribution, we discuss—based on studies about thin organic surface layers of photoreactive organosilane bearing *o*-nitrobenzyl esters (Mol-1)—three *ex situ* methods to correctly recognize the surface terminations without requiring a topographical reference. Further, considering time dependent effects, we present a time dependent *ex situ* FFM measurement, in which a well-defined surface pattern using four different illumination times can be inscribed and then clearly visualized by FFM to reveal the photogenerated surface changes. Utilizing the illumination system of the optical microscope attached to the AFM and employing the AFM cantilever as a bar-shaped non-contact mask, we finally developed a novel *in situ* illumination FFM experiment which enables the detection of real height and real-time surface changes during illumination. Employing this *in situ* FFM measurement procedure, time dependent effect of the surface modification during the photoreaction can be revealed. For this study, we used a photoreactive polymer (polynorbornene with pendant spiropyran groups).

## Materials and Methods

### Materials

**Figure 1:**
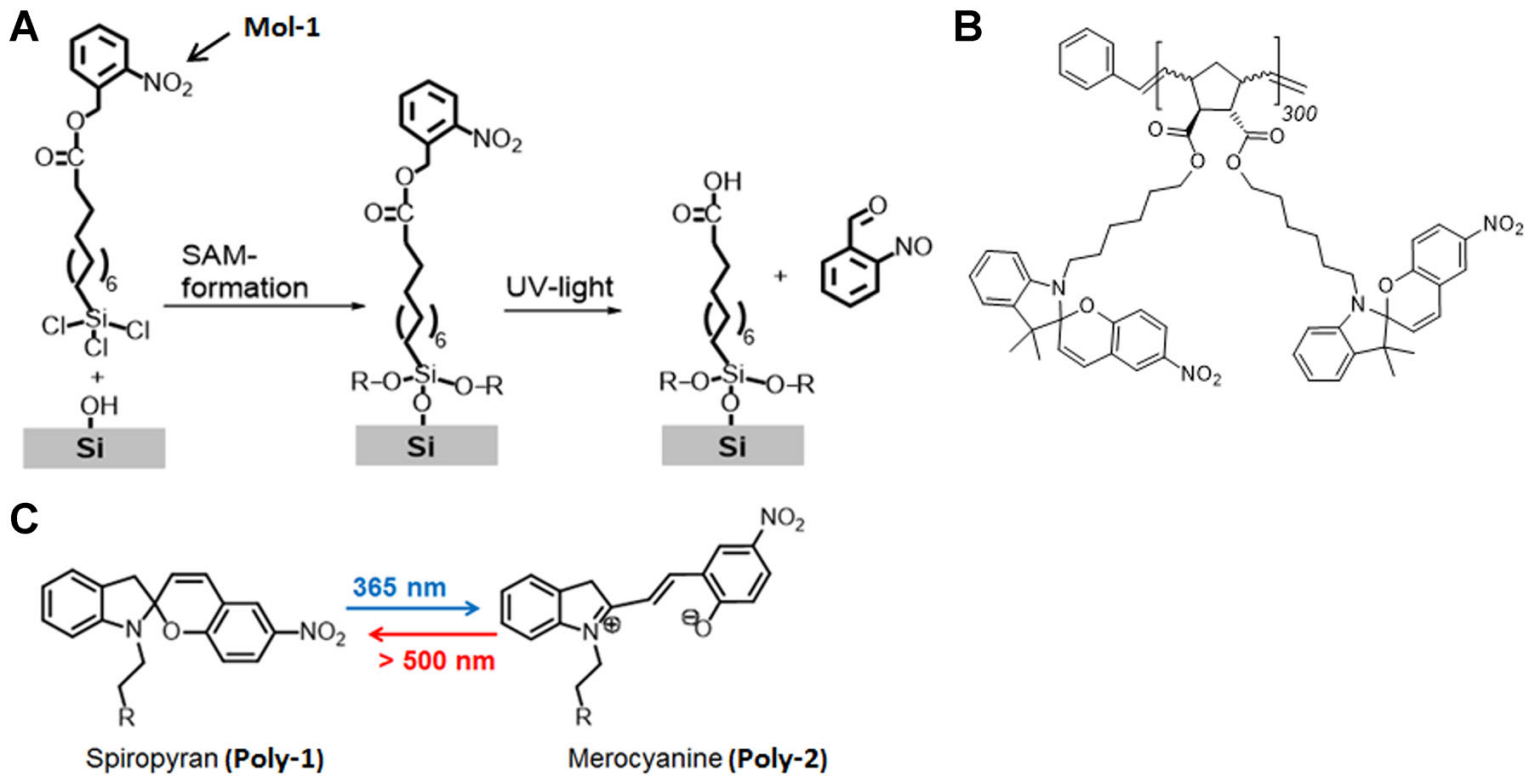
Photoreaction pathway and products of (A) organosilanes bearing *o*-nitrobenzyl esters endgroup (Mol-1) and (B) polynorbornene bearing spiropyran side groups(Poly-1) investigated by FFM. The reversible photoreaction of polynorbornene bearing merocyanine side groups (Poly-2) within *in situ* illumination FFM measurements is demonstrated in (C) by the red arrow.

The first thin layer, characterized by FFM measurements is a 2D layer of bifunctional molecules bearing photoreactive ester groups, capable of undergoing a photocleavage reaction. The bifunctional molecule *nitrobenzyl 11-(trichlorosilyl) undecanoate* (Mol-1, see Fig. 1A) is based on a trichlorosilane head group and a photoreactive *o*-nitrobenzyl ester tail group. Using *o*-nitrobenzyl ester groups the occurring deprotection upon irradiation with UV-light leads to the associated formation of the photoproduct (Reichmanis *et al*., 1985; Ryan *et al*., 2004; Critchley *et al*., 2006; Yamaguchi *et al*., 2008; Prompinit *et al*., 2009; Driscoll *et al*., 2010). For the *in situ* illumination FFM measurement polymer films of *poly[(±)- endo,exo-bis(6-(3′,3′-dimethyl-6-nitrospiro[chromene-2,2*'*-indolin]-1*'*-yl)hexyl)-bicyclo[2.2.1]hept-5-ene-2,3-dicarboxylate]* (Poly-1, see Fig. 1B) are used. Spiropyranes are characterized by their well-known reversible photochromism. In a first irradiation step, the switch from the apolar spiropyran form (Poly-1) to the zwitterionic merocyanine form (Poly-2) is induced via UV light (365 nm). In a second illumination step (*in situ*), the spiropyran is reformed using the visible light regime (see Fig. 1C) (Heiligman-Rim *et al*., 1962; Crano and Guglielmetti, 2002; Hauser *et al*., 2012).

The photocleavage reaction of Mol-1 as well as both pathways of the reversible photoconversion of the polymeric layer were controlled by Fourier Transform Infrared (FTIR) Spectroscopy, respectively. Detailed information of synthesis and the photoreaction is provided in the supporting information 2(a, b, c).

For the preparation of the photoreactive organosilane layers clean Si/SiO_2_ substrates were immersed in a solution of 2-nitrobenzyl 10-(trichlorosilyl) undecanoate in pure toluene (7 mM) for 1 h. The formation of an oligolayer based on the reactivity of trichlorosilanes forming 3D networks has to be considered (Silberzan *et al*., 1991). The spiropyran polymer was spin cast from toluene solutions (10 mg ml^−1^ onto Si substrate).

### Methods

FFM is a contact mode AFM technique, where the AFM cantilever is scanned across the surface perpendicular to its long axis. The resulting lateral torsion of the cantilever, induced by the friction force acting on the tip, is recorded by the feedback laser and a four-quadrant photodiode (Marti *et al*., 1990). Using FFM, the material contrast on the surface can be detected (Hlawacek *et al*., 2009), and corresponding micro-scale friction coefficients can be determined (Kluensner *et al*., 2010). Our FFM measurements were performed with an Asylum Research MFP3D AFM under ambient conditions using soft TiN coated silicon probes (CSG01 from NT-MDT). These probes, which are specially designed for friction force measurements, are a rectangular beam cantilever (nominal length: 350 µm; width: 30 µm; thickness: 1 µm) with a typical force constant of ca. 0.03 N/m. Due to the TiN coating, the curvature radius of the tip is above 35 nm. To reduce topographical artifacts in the resulting friction data, all the friction images have been processed as “trace minus retrace” images, so called “real friction” images, which are obtained by the following calculation: real friction force = (FFM trace − FFM retrace)/2 (Kalihari *et al*., 2010). FFM is particularly sensitive to friction changes, which strongly depend on the interaction between the tip and the terminating group of the molecules within the photoreactive patterned surface (Hlawacek *et al*., 2009).

### *Ex situ* Illumination Experiments

The *ex situ* irradiation experiments of a thin liquid film of Mol-1 were carried out with a medium pressure Hg lamp (100 W, from Newport, model 66990) equipped with a filter for the range of >300 nm. In these experiments, the light intensity at the sample surface was measured with a spectroradiometer (Solatell, Sola Scope 2000TM, spectral range from 230 to 470 nm) and calculated to be 19.8 J cm^−2^ for >300 nm [see Supporting Information 3(a)]. Comparing the FTIR spectra of a thin liquid film of Mol-1 before and after illumination [see Supporting Information 2(a)], the progress of photocleavage reaction is determined. All UV illuminations were carried out under inert gas atmosphere (N_2_). *Caution: UV irradiation causes severe eye and skin burns. Precautions (UV protective goggles, gloves) must be taken!*

### *In situ* Illumination FFM Measurements

In a first illumination step, a thin liquid film of Poly-1 was performed with a medium pressure Hg lamp (100 W, from Newport, model 66990) equipped with a filter for the range of 350–450 nm. In these experiments, the light intensity at the sample surface was measured with a spectroradiometer (Solatell, Sola Scope 2000™, spectral range from 230 to 470 nm) and calculated to be 8.4 J cm^−2^ [see Supporting Information 3(b)]. Comparing the FTIR spectra of Poly-1 sample before and after illumination [see Supporting Information 2(b)], the progress of “photoreaction: spiropyranes to merocyanine” is determined. All UV illuminations were carried out under inert gas atmosphere (N_2_).

The second illumination step for the *in situ* FFM measurements was performed on an Asylum Research MFP3D AFM system. The halogen lamp, which is utilized for optical imaging of the sample surface within the AFM, is replaced by a Xe 150 W arc lamp. Using an optical fiber, the light from the Xe arc lamp is transmitted through the optical path of the AFM head and the prism of the cantilever holder to the opaque cantilever and focused in a spot of about 2 mm in diameter. The light through the AFM's optical system, which is transparent in the range from 380 to 900 nm, is recorded by a photodetector in the AFM setup [see Supporting Information 3(c)]. The bar-shaped CSG01/TiN cantilever is employed as a noncontact mask resulting in a rectangular shadow on the surface, which can be further investigated by FFM. Comparing the FTIR spectra of Poly-2 sample before and after illumination [see Supporting Information 2(c)], “the reversibility of this photoreaction: merocyanine to spiropyranes” is determined.

## Results and Discussions

### *Ex situ* Identification

In the following, we present three FFM-based methods, which allow for *ex situ* identification of illuminated and non-illuminated areas on the same organosilane sample by illumination through different types of masks.

#### Method 1

**Figure 2:**
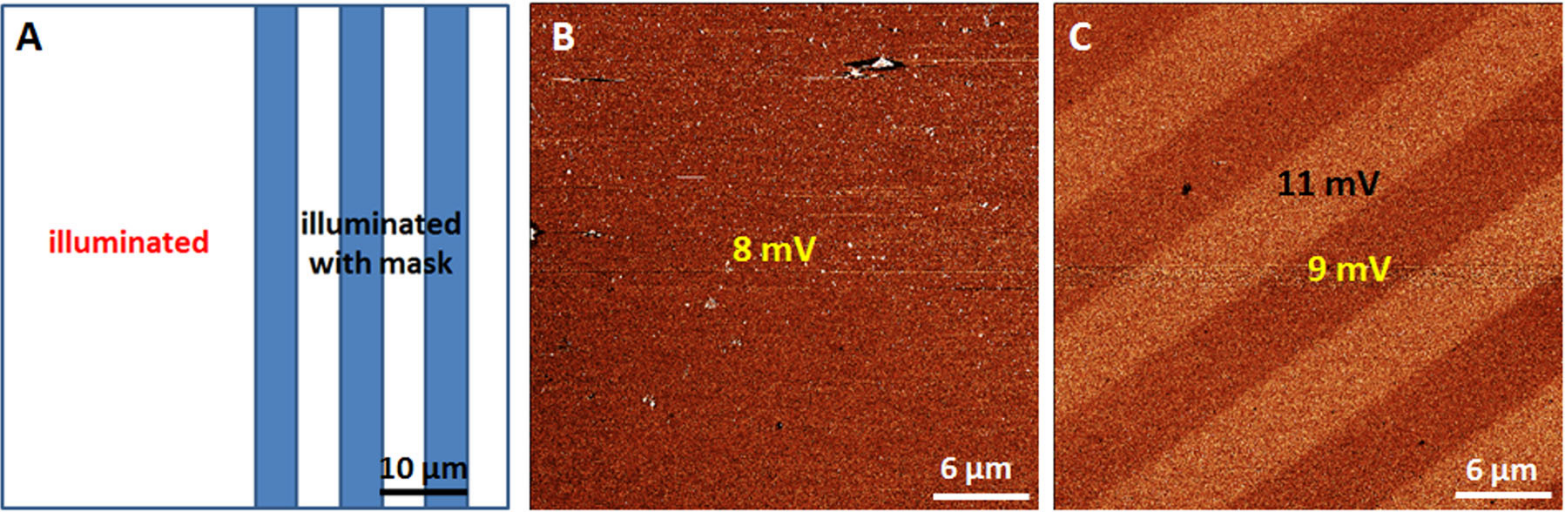
Illustration and results of method 1: (A) Scheme of Mol-1 sample illumination, the right half is illuminated through a contact mask with a 10 µm pitch (blue stripes). (B) Thirty micrometer FFM image (*z*-scale: 5 mV) of the completely illuminated area in the left half of the sample and (C) 30 µm FFM image (*z*-scale: 5 mV) of the patterned sample.

In the first procedure, a sample is examined where one half is completely illuminated and the other half of the sample is illuminated using a stripe contact mask. The scheme is presented in Figure 2A. The resulting left part of the sample is completely photoreacted to a uniform featureless surface without showing a friction contrast (see Fig. 2B). The right half is illuminated through a stripe mask with 5 µm lines and 5 µm spaces. The friction image presented in Figure 2C shows a discernible stripe pattern with the expected 5 µm spacing. In all friction images presented hereafter, bright areas mean higher friction and dark ones correspond to lower friction. The friction signals in Figure 2B and C are given in volts corresponding to the cantilever torsion detected by the four-quadrant photodiode. Since we are only interested in a semi-quantitative differentiation of the adhesion, no transformation into surface contact forces and friction coefficients has been performed. By comparison with the friction value of the completely illuminated area, the surface pattern of the right half can be identified: The low-friction stripes correspond to the illuminated surface layer of Mol-1 and the high-friction stripes are the pristine non-illuminated areas. Because these two halves cannot be measured simultaneously in one scanning process, a slight friction shift between both sample areas is obtained, which is attributed to a change in the tip-sample interaction while measuring. Accordingly, this method is appropriate for clean and rather smooth SAM surface, which prevent the change of tip geometry induced by surface contamination during the measurement.

**Figure 3:**
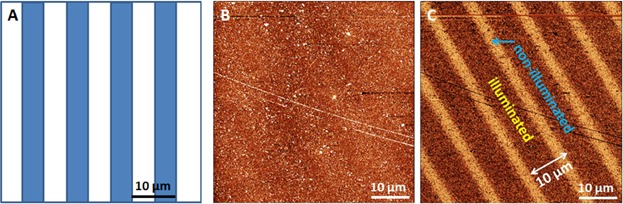
(A) Mol-1sample illuminated through a contact mask with 10 µm pitch. (B) Fifty micrometer topographic (*z*-scale: 5 nm) and (C) corresponding FFM (*z*-scale: 5 mV) images of the stripe pattern.

However, the photolithographic processes using symmetric contact stripe masks with same line spacing do not always produce a symmetric stripe pattern on the surface. An extreme situation is demonstrated in Figure 3. Illuminated through a stripe mask with 5 µm lines and 5 µm spaces for longer times, the FFM image revealed stripes of about 3 and 7 µm widths (see Fig. 3C), which are also—but less clearly—visible in the corresponding topographic image (see Fig. 3B). Due to light scattering (Thompson *et al*., 1994) the illuminated areas are broader than the non-illuminated ones.

#### Method 2

Obviously, using a mask with different stripe width for illuminated and non-illuminated areas is an easiest way to identify the patterned surface. However, employing stripe mask with same line spacing, the following method could be considered.

**Figure 4:**
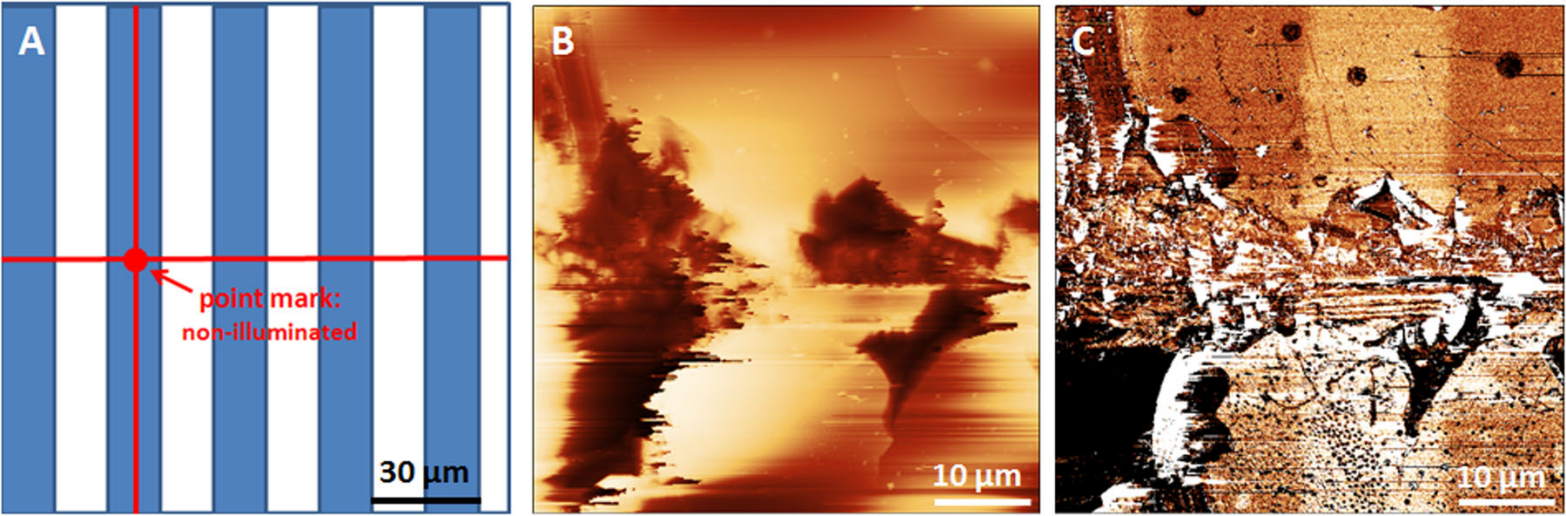
Illustration and results of method 2: (A) Scheme of Mol-1 sample illumination through a contact mask with 30 µm pitch and a cross mark in the non-illuminated area. (B) Topographic (*z*-scale: 5 nm) and (C) corresponding FFM (*z*-scale: 5 mV) images of 15 µm stripe pattern with cross mark.

Two crossed lines are engraved on the sample to mark a reference point, which can be exactly covered by the stripe mask with 15 µm lines afterwards (see Fig. 4A). While the topographic image presented in Figure 4B shows an enormous surface damage due to the cross marking, the corresponding friction image in Figure 4C allows a clear identification of the stripe pattern by means of the marked point, which allows to locate the non-illuminated stripe after contact lithography despite the surface damage. The high friction stripes correspond to the non-illuminated ones. As a result of tremendous abrasion due to the engraving, the FFM sensitivity to different chemical surface components has been reduced. Consequently, a more gentle surface marking method is recommended when applying this procedure.

#### Method 3

**Figure 5:**
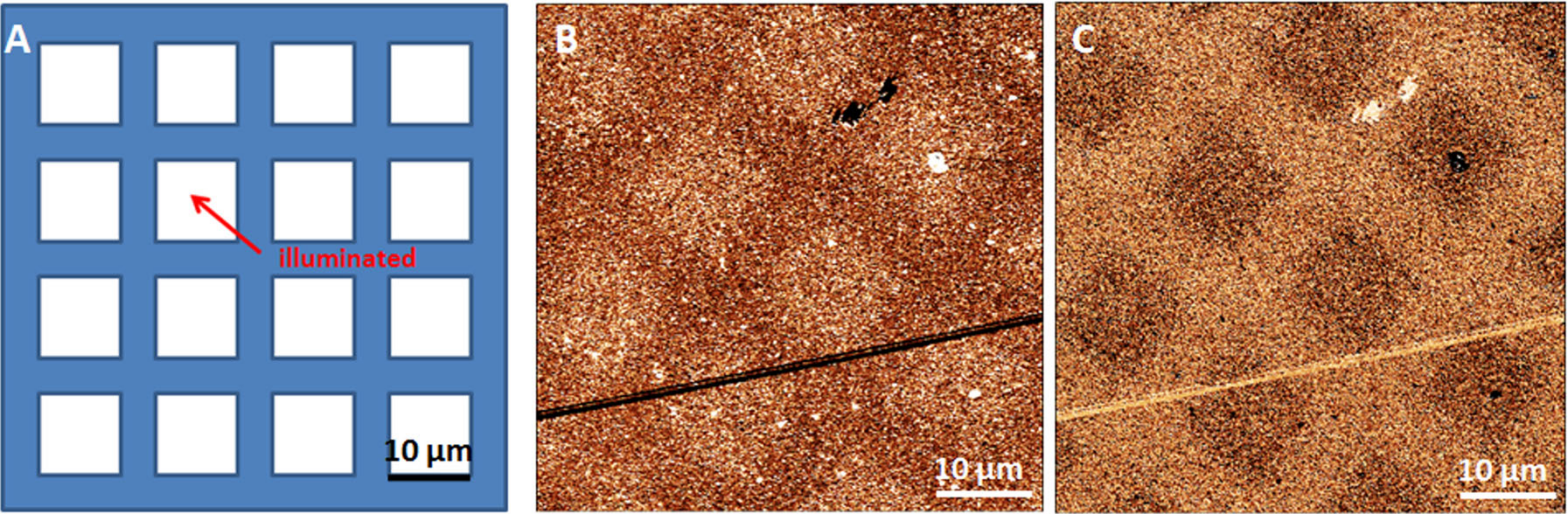
Illustration and results of method 3: (A) Illumination schema using a TEM grid (blue grid). (B) Fifty micometer topographic (*z*-scale: 5 nm) and (C) corresponding FFM (*z*-scale: 5mV) images of a terminated grid pattern inscribed in a surface layer of Mol-1.

In this approach, the illuminated and non-illuminated areas are of different geometry and thus can be easily identified by shape of the found surface structures. For this, the sample was illuminated through a contact mask with a two-dimensional grid structure. Using a transmission electron microscope (TEM) grid as contact mask (see Fig. 5A), the illuminated square areas with low friction can be easily detected and recognized in the friction image (see Fig. 5C). Figure 5B presents the corresponding topographic FFM image, which shows a slight height increase of the illuminated terminations. As both the topography and the friction image reveal, the direct contact masking causes surface damage as scratching and contamination. Thus, a photolithography process with a non-contact mask near the surface is desired and will be discussed later.

These *ex situ* FFM methods described above allow the identification of the illuminated and non-illuminated areas on the patterned surface in friction images without requiring the detection of a height difference in the topographical image. As a result of the reduced friction force of the photoproducts formed after illumination, the further detection of time dependent effects during surface photoreaction becomes possible as will be described in the following.

### Time Dependent *ex situ* Identification

**Figure 6:**
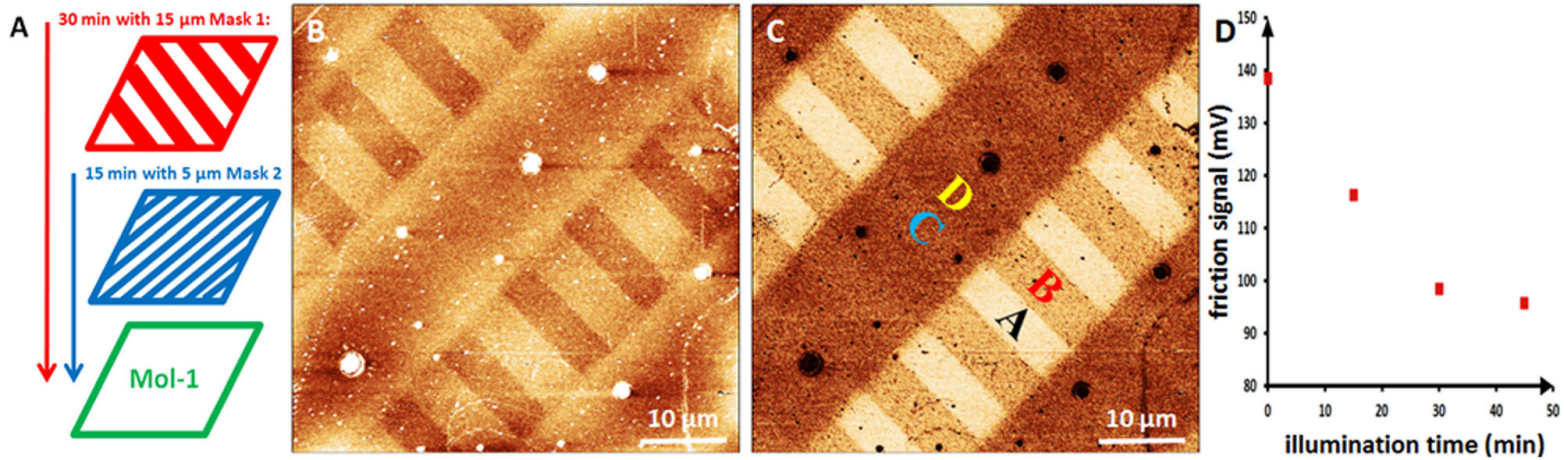
Time dependent *ex situ* FFM measurements on surface layer of Mol-1: (A) Scheme of sample preparation: 30 min illumination through mask 1 with 30 µm pitches; second illumination (15 min) through mask 2 with 10 µm pitch. (B) Resulting topographic (*z*-scale: 5 nm) and (C) corresponding FFM (*z*-scale: 5 mV) images from the multistage patterned film. The respective illumination times are indicated in the FFM image (A: 0 min; B: 15 min; C: 30 min; D: 45 min). (D) Dependence of the average friction signal on the illumination time.

In a first approach towards time dependent investigation, this *ex situ* FFM measurement has been performed as demonstrated in Figure 6. In this experiment, the surface layer of Mol-1 has been exposed twice by UV light using crossed stripe masks. First, the sample surface was illuminated for 30 min through mask 1 with 15 µm lines and spaces. The resulting intermediate pattern consists of alternating 15 µm non-illuminated and 15 µm illuminated stripes. In the second step, the patterned surface is illuminated for 15 min through mask 2 with 5 µm stripes, which has been oriented perpendicular to mask 1. Both, the non-illuminated as well as the 30 min illuminated stripes are partly converted into alternating patches with a total illumination time of 15 and 45 min, respectively. Figure 6B and C present the final surface morphology and friction patterns, respectively. The friction image shows a clear pattern of regular 15 µm × 5 µm patches with four different friction levels, which are corresponding to four different conversion times and surface properties.

As obtained in the *ex situ* measurements, the friction force decreases after illumination. Therefore, the patches with the highest friction level correspond to the non-illuminated termination (labeled A in Fig. 6C). According to the lateral layout of the patch pattern, their neighboring patches in the alternative 15 µm stripes—formed in the first step—can be identified as the 30 min illuminated terminations (labeled C), which shows second lowest friction level. Similarly, the neighboring patches of A in the adjacent 5 µm stripes—formed in the second step—can be identified as the 15 min illuminated surface patch (labeled B), which show the second highest friction level. The remaining patches with the lowest friction level are the 45 min illuminated terminations (labeled D). On this patterned surface, four different surface areas caused by four different illumination times have been identified and hierarchically ordered by their friction levels. The sensitivity of this time dependent *ex situ* FFM measurement is sufficient for detecting slight friction shifts, which is in agreement with the change of the tip-sample interaction induced by illumination. The confirmed sequence of friction level A > B > C > D reveals the relationship between friction signal and illumination time. In Figure 6D, the friction signal is plotted as a function of the cumulative illumination time. The sequence of the friction signals correspond to the illumination time and thus can be related to the yield of the photoreaction. Figure 6D shows that a strong decrease in friction induced by surface photoreaction occurred in the first 30 min of illumination. To detect the details of friction shift in the first 30 min, this time dependent *ex situ* measurement has to be repeated with shorter illumination intervals. However, only four time intervals per one time dependent *ex situ* measurement can be obtained with this procedure. Thus, there is a demand for a true *in situ* investigation procedure.

### *In situ* Identification: Real-Height and Real-Time Detection

For both, real-height and real-time detection (recording of the friction changes under continuous illumination), a specific *in situ* FFM experiment has been designed, which differs in two aspects from *ex situ* measurements reported so far. First, as mentioned before, the small glass prism on the tip holder at the end of optical path in the AFM system shields the light with wavelength smaller than ca. 380 nm. Thus, the former Mol-1 terminated surface layer, which undergoes a photoreaction only with light in the UV-range, is not suitable for the following *in situ* FFM measurements. In order to overcome this device limitation, we used alternative surface layers composed of a polynorbornene bearing merocyanine side groups (Poly-2, see Fig. 1B), which can reversibly photoreact to spiropyran moieties with light of wavelengths >500 nm. According to the FTIR spectroscopy results [see Supporting Information 2(c)], a photoconversion of the merocyanine into the spiropyran with the output light throughout the AFM can be clearly determined. Thus, the *in situ* illumination FFM experiment has been performed on polynorbornene with merocyanine side groups.

**Figure 7:**
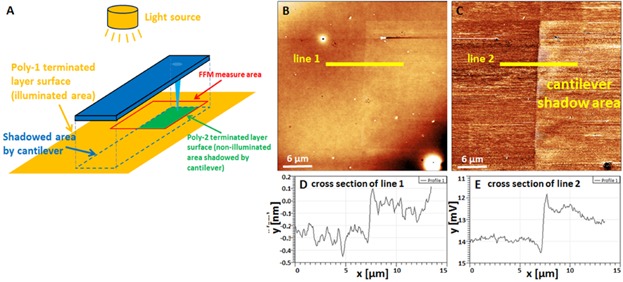
Principle of *in situ* irradiation in the AFM: (A) A bar-shaped AFM cantilever is put as a noncontact mask over a merocyanine (Poly-2) terminated polynorbornene film surface, which is illuminated in AFM. (B) Thirty micrometer topographic (*z*-scale: 10 nm) and (C) corresponding FFM (*z*-scale: 5 mV) images of shadow area covered with a stationary AFM cantilever (lower right in both images). The corresponding cross section in the topography (D) and friction images (E) reveal clear morphological and friction changes after illumination in the AFM system.

Second, *ex situ* FFM characterization on the illuminated patterned surface is often accompanied by surface abrasion and contamination induced by using a contact mask, which is likely to affect the accuracy of further friction force measurements. For avoiding those undesirable surface changes during illumination, a commercial bar-shaped AFM cantilever is employed as noncontact mask. Figure 7 demonstrates the proof of principle of the *in situ* irradiation and FFM experiments. Here, the polynorbornene with merocyanine terminated thin film was illuminated through the prism for 30 min without moving the 30 µm wide CSG01/TiN cantilever, which was about 25 µm above the surface (see Fig. 7A). Figure 7B shows a subsequently scanned 30 µm × 30 µm topography image of an area that has been shadowed in the right by the upper left corner of the cantilever, the corresponding FFM image is shown in Figure 7C. Both images clearly reveal the rectangular non-illuminated area, which has been shadowed by the cantilever. Cross sections along lines 1 and 2 (see Fig. 7D and E) reveal a reduction of layer height and friction due to the photoreaction. We want to note here that the real height change induced only by illumination of about 0.3 nm can be used as a reliable height reference for more precise identifications of photopatterned films.

**Figure 8:**
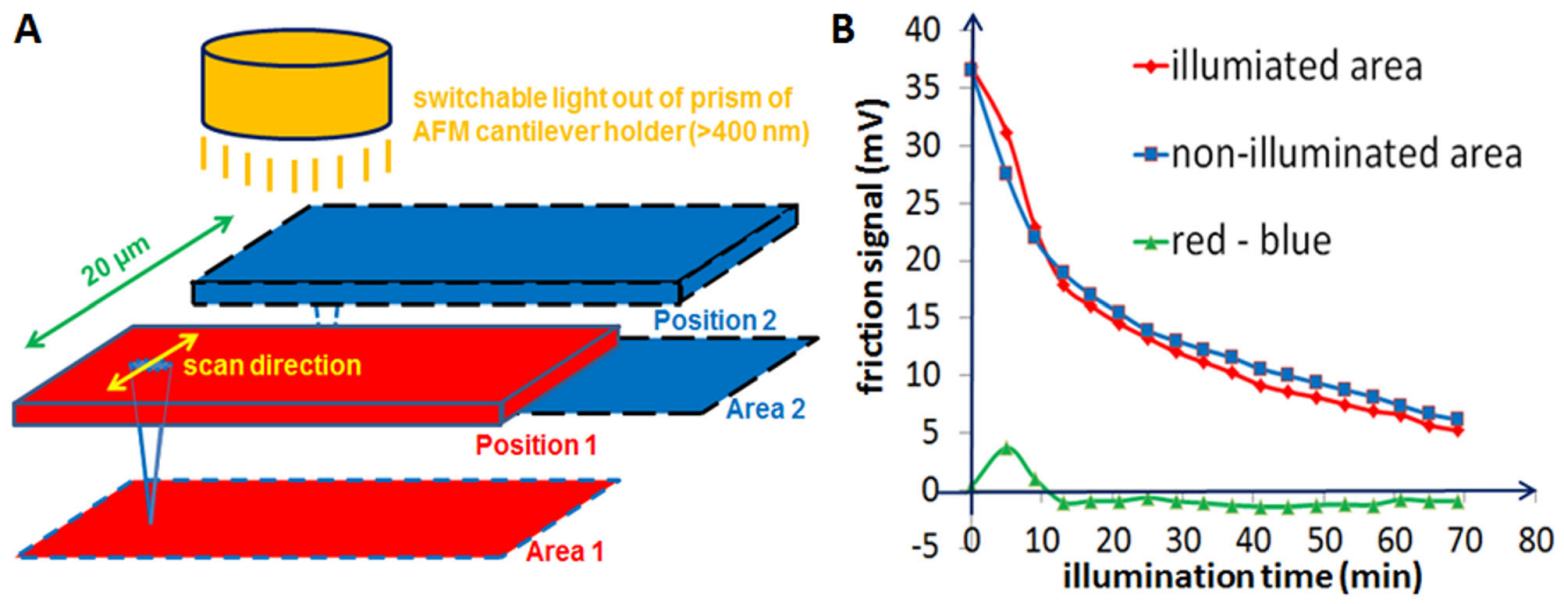
Scheme of *in situ* FFM measurement model setup: (A) An AFM cantilever is scanned alternately on two measurement areas (illumination position 1 and reference position 2) on a merocyanine (Poly-2) terminated polynorbornene film surface upon illumination in FFM contact mode. (B) Relationships between friction force and illumination time in illumination (red curve) and reference area (blue curve), respectively. The green curve, which represents the friction change only induced by visible light illumination, is obtained by the difference of red curve (illuminated) minus blue curve (non-illuminated) at the corresponding illumination time. The highest standard deviation of friction values in the green curve is of 0.15 mV.

Figure 8 presents now the scheme and results of our true *in situ* illumination and FFM measurements. A CSG01/TiN cantilever is put over the Poly-2 terminated surface. Friction force measurements alternate between the illumination area 1 and the reference area 2 (see Fig. 8A). First, the AFM probe is scanned in both areas without illumination to record the original friction force of non-illuminated area 1 and 2. Second, while the probe stays over area 2, light is turned on. Area 1 is illuminated for 5 min, and area 2 is simultaneously shadowed by the cantilever. In the third step, light is turned off and the probe is scanned across both areas again. In the subsequent measurement procedure, once the scans in both areas are finished, the probe is moved to area 2. The light is then turned on for 4 min. The third step is repeated until the illumination time accumulates to 69 min in area 1. Obviously, in these series of FFM measurements, the area 1 is continuously illuminated and the area 2 is always covered by the cantilever. In Figure 8B, all plotted friction force values in the red (or blue) curve are average values, which are calculated by the friction forces in three different positions of area 1 (or area 2). The red and blue curves, respectively present the friction change in the illumination area and non-illumination area. The green curve is obtained by “subtraction: red curve minus blue curve” with a highest standard deviation of 0.15 mV, which is roughly equal to 25% of the smallest value (−0.6 mV) of green curve.

In comparison with the *in situ* AFM measurement by Terrettaz *et al*. (1998), which focused on the investigation of morphological changes as a function of time, the characterization parameters of our *in situ* FFM measurements are in particular the photoinduced changes of surface friction beside changes in height. However, we have to note that friction force in contact mode AFM, as a measurement parameter, is easily influenced by the artifacts due to repeated scanning in the same position. During the continuous scanning at the same position, some material is deposited on the tip apex and the tip shape can also suddenly change due to surface contamination. This becomes clear by the reduction of the friction signal in non-illuminated area 2 (see blue curve in Fig. 8B), which is induced by the repeated scanning process in same area or maybe slight conversion of merocyanine group with the assistance of frictional heat. These artifacts cannot be neglected also in the illuminated area. The friction change induced by visible light illumination is revealed by the green curve, which is created by subtracting the blue curve (non-illuminated) from the red curve (illuminated) in order to eliminate the artificial friction changes. Therefore, this green curve represents the real-time photogenerated friction change. Our *in situ* FFM investigation reveals an initial increase compared to the non-illuminated area, whereas after 13 min the already in Figure 7 observed reduction in the friction signal is found. Further illumination does not change this situation. The unexpected increase in the friction signal after the first illumination step might be due to the formation of an intermediate photoproduct of merocyanine–spiropyran photoreaction (Wojtyk *et al*., 2001).

## Conclusions

In this contribution, we have demonstrated the power of FFM to identify modifications of photoreactive organic surface layers. In particular, we have evaluated three *ex situ* FFM characterization methods, which allow easy identification of different surface terminations on photogenerated organosilane surface patterns. By using half masking, surface marking and grid mask, the *ex situ* photogenerated patterns can be recognized even when the height contrast in the topographical image is invisible. Comparatively, the method using a grid mask is the most easy and appropriate way.

With the help of the above mentioned friction reference, time dependent *ex situ* identification becomes possible. An organosilane surface pattern consisting of the same surface termination can be formed with different illumination times. The friction changes can subsequently be detected in the friction image, and a relation between friction signal and illumination time can be established for a maximum of 4 illumination times. After ca. 30 min illumination, the photoreaction on the surface terminations, which corresponds to the friction change, has almost saturated.

An FFM procedure using the AFM cantilever as noncontact mask has been designed and first tested. The real height (ca. 0.3 nm) and real friction change induced by *in situ* irradiation are detected in both, the topographic and the friction image of the polymer film. The real height and friction reference make the identification of photogenerated pattern more precise. Moreover, the developed *in situ* illumination FFM measurement allows determining details of the surface modification during the photoreaction. The method indicated for the merocyanine–spiropyran photoreaction hints to the existence of an intermediate photoproduct in the early stage of the photoreaction. Future *in situ* FFM measurements with better time resolution and replacing the glass prism by a quartz one to achieve full wavelength illumination may clarify this assumption.
