# Supplementary material for: Ex situ and in situ characterization of patterned photoreactive thin organic surface layers using friction force microscopy
Source: Scanning. 2014 Sep 2;36(6):590–8. doi: 10.1002/sca.21159 (PMC4286208; doi:10.1002/sca.21159)
Supplement: Supplementary file 1 — Supporting Information. [file sca0036-0590-sd1.doc]

**Supporting Information**

**1. Synthesis of the photoreactive materials**

**a) Synthesis of 2-nitrobenzyl 11-(trichlorosilyl)undecanoate (Mol-1)**

The photosensitive bifunctional molecule was synthesized in a two step reaction. To exclude light from the reaction aluminium foil was used.

In a first step 2.0 g (9.9 mmol) of undec-10-enoyl chloride was added to a solution of 1.68 g (10.9 mmol) of (2-nitrophenyl) methanol, dissolved in a mixture of 0.88 ml (10.9 mmol) of pyridine and 40 ml of dichloromethane. To exclude light from the reaction aluminium foil was used. The reaction was stirred for 48 h at ambient temperature until a complete conversion was observed. The organic layer was extracted with 3 x 20 mL of 5% hydrochloric acid solution to remove excess pyridine and 20 mL of saturated sodium bicarbonate solution, then dried over sodium sulphate. The solvent was removed in a vacuum and subsequently a column separation using cyclohexane/ethyl acetate (8:1) for product purification was performed.

**Yield:** 2.63 g of a white solid (83 % of theoretical yield).

**^1^H-NMR:** (δ, 400 MHz, 20°C, CDCl_3_): 8.05 (dd, 1H, ph^3^); 7.62 (t, 1H, ph^4^); 7.57 (d, 1H, ph^6^); 7.46 (t, 1H, ph^5^); 5.76 (m, 1H, =CH-); 5.48 (s, 2H, O-CH_2_-ph); 4.91 (dd, 2H, CH_2_=); 2.38 (t, 2H,-C2-(COO)); 2,00 (q, 2H,C9); 1.63 (quint, 2H,C3); 1.27 (m, 10H,C4-C8) ppm.

**^13^C-NMR:** (δ, 125 MHz, 20°C, CDCl_3_): 172.30 (1C, COO); 147.56 (1C, ph^2^-NO_2_); 137.13 (1C, C10(C=C)); 135.20 (1C, ph^1^); 134.24 (1C, ph^5^); 128.36 (2C, ph^4,6^); 125.67(1C, ph^3^); 115.15 (1C, C11(C=C)); 63.76 (1C,(COO)-CH_2_-ph); 34.05 (1C,C9); 34.00 (1C,C2); 29.27 (1C,C8);29.18 (1C,C7); 29.07 (1C,C6); 29.04 (1C,C5); 28.87 (1C,C4); 24.93 (1C,C3) ppm.

In a second step 1.0 g (7.4 mmol) of trichlorosilane was added to a solution of 1.0 g (3.1 mmol) of (2-nitrobenzylundec-10-enoate, dissolved in 5 ml of dichloromethane. As catalyst hexachloroplatinic acid was used. The reaction was stirred for 24 hrs. at ambient temperature until a complete conversion was observed.

**Yield:** 1.38 g of a white, yellowish liquid (97.3% of theoretical yield).

**^1^H-NMR:** (δ, 400 MHz, 20°C, CDCl_3_): 8.05 (dd, 1H, ph^3^); 7.59 (t, 1H, ph^4^); 7.53 (d, 1H, ph^6^); 7.43 (t, 1H, ph^5^); 5.48 (s, 2H, (O-CH_2_-ph)); 2.35 (t, 2H,-C2-(COO)); 1.61 (m, 2H,C3); 1.51 (m, 2H,C10); 1.35 (quint, 2H,C11); 1.28 (m, 12H,C4-C9) ppm.

**^13^C-NMR:** (δ, 125 MHz, 20°C, CDCl_3_): 173.12 (1C, COO); 147,64 (1C, ph^2^-NO_2_); 133.61 (1C, ph^6^); 132.23 (1C, ph^1^); 129.1 (1C, ph^4^); 128.73 (1C, ph^5^); 125.01 (1C, ph^3^); 62.76 (1C,(COO)-CH_2_-ph); 34.13 (1C,C2); 31.75 (1C,C9); 29.32 (1C,C8); 29.23 (1C,C7); 29.15 (1C,C6); 29.07 (1C,C5); 28.94 (1C,4); 24.84 (1C,C3); 24.27 (1C,C11); 22.23 (1C,C10) ppm.

**IR-Data** **(CaF_2_, cm^-1^):** 2925; 2853; 1742; 1613; 1578; 1528; 1447; 1343.

**2. FTIR spectra of both photoreactive systems**

**a) FTIR spectroscopy results of a thin liquid film (Mol-1) before and after illumination**

Comparing the FTIR spectra of a thin liquid film of Mol-1 prior to and after illumination (see Figure 9), the depletion of peaks at 1740 cm^-1^, which can be attributed to the ester C=O, and peaks at 1528 cm^-1^ and 1340 cm^-1^, assigned to the nitro group, can be detected. A weak new signal that emerges at 1706 cm^-1^ is representative for the formation of the carboxylic acid group as photoproduct.

**b) FTIR spectroscopy results of the spiropyrane (Poly-1) terminated sample in first illumination step (before and after illumination)**

In first illumination step, spiropyranes (Poly-1) undergo a photoreaction to the open merocyanine (Poly-2) form with UV-light of 350-450 nm (see Figure 10).

FTIR measurements of irradiated samples of Poly-1 showed three main differences of the spiropyran and the merocyanine form. After UV Illumination a broadening of the peak at 1276 cm^-1^, representative for the building of the new C=N^+^ bond and two more peaks at 1422 cm^-1^ (formation of the C-O- bond) and at 1595 cm^-1^ (C=C double-bond in the center of the molecule) are observed.

**c) FTIR spectroscopy results of the merocyanine (Poly-2) terminated sample in second illumination step (before and after illumination)**

In second illumination step, merocyanines (Poly-2) undergo a reversible photoreaction to spiropyran (Poly-1) with visible light (see Figure 11).

After in-situ illumination with visible light (Xe Lamp) in AFM, a reduction of the peaks characterizing the formed merocyanine form can be observed and the spiropyran ring system reappears demonstrating the reversibility of this photoreaction.

**3. Light spectra in both ex-situ and in-situ illumination experiments**

**a) Solatell spectroradiometer spectra of a medium pressure Hg lamp (100 W, from Newport, model 66990) equipped with a filter for the wavelength of >300 nm for thin liquid film (Mol-1) in ex-situ illumination experiments (see Figure 12)**

**b) Solatell spectroradiometer spectra of a medium pressure Hg lamp (100 W, from Newport, model 66990) equipped with a filter for the wavelength 350-450 nm for spiropyrane (Poly-1) terminated sample in the first illumination step (ex-situ illumination) (see Figure 13)**

**c) Normalized spectra of light throughout AFM system on merocyanine (Poly-2) terminated sample in the second illumination step (in-situ illumination) using a photodetector in the AFM setup (see Figure 14)**
